# Notes and News

**Published:** 1890-11-22

**Authors:** 


### Notes and News.

Collected and Communicated.

At the annual meeting of the Birmingham Town Council it was announced that Mr. George Frederick Muntz has signed a deed of trust, in which it is declared that the securities of the nominal value of £21,000 are vested in "the official trustees of charitable funds," and assigning the annual income therefrom, £600, to be distributed yearly by the trustees among such of the charitable institutions of the City of Birmingham, for the medical and surgical relief or alleviation of sickness, or bodily infirmities, but excluding hospitals or asylums for mental diseases, and institutions or houses for convalescents.

The Royal Isle of Wight Infirmary held its annual meeting on November 5th, and added by acclamation to its title the words, "and County Hospital." The committee have applied a portion of the £500 for "Building and Furnishing," announced in last annual report, by building a new ward, a nurses' room, enlarging the dispensary, and other necessary alterations, which will enable them to open six additional women's beds. The ward will be ready for occupation when furnished. There are also four bath-rooms being fitted. The number of in-patients for the year was 417; out-patients, 1,218. It was announced that four more free beds would in future be available at the Convalescent Home.

At a meeting of the Metropolitan Asylums Board, on Saturday, it was reported that the total increase of the number of fever patients under treatment in the hospitals of the Board was 102. There are now 2,068 patients suffering from scarlet fever, and 160 from diphtheria. Altogether there are 2,435 patients in the hospitals. During the past fortnight 60 of the patients had died, and 302 had been discharged as recovered. The number of cases notified under the Notification of Diseases Act during the fortnight was 1,851, of which 938 were of scarlet fever and 344 of diphtheria. It was stated that there had not been a case of small-pox in the hospitals of the Board for the past five weeks, and the chairman of the Small-pox Committee stated in answer to the chairman of the Board that he was backed up in his opinion by the Local Government Board in a report that the immunity of London from small-pox was wholly due to the small-pox ships and hospitals of the Board and the system of isolation.

The tenth annual report of the Notre Dame Hospital at Montreal tells of 1,716 in-patients of whom 500 were paying or private patients. The report is a very full one and gives an interesting account of the hospital. Notre-Dame Hospital was established for the cure and relief of the sick, without distinction of race or creed. The nursing of the patients is entrusted to the reverend Grey Nuns, nineteen of whom reside continually in the Hospital. In addition to these, two ladies of the same community come to the hospital every evening, to watch the sick during the night. The hospital is blessed with an ambulance and with the electric light and all modern improvements. The death-rate is 5·6 per cent.

Christmas parcels have been received here from Woolwich and from Nurse Elizabeth Bishop; at Norfolk House from Miss Lockyer, of Marlborough College. We have also to acknowledge a parcel from Mrs. William Black. Parcels received one week will be acknowledged in the next week's Hospital. They should be addressed "Christmas Parcel, the Hospitals Association, Norfolk House, Norfolk Street, Strand, London," and will be gladly received up till December 17th. We hope many nurses will enter for the bed-jacket competition, of which particulars were given in our issue of November 8th, as all these garments will be added to the Christmas parcels.

A whole batch of reports come from Australia, and some of them are worthy of study. The Port Fairy Hospital and Benevolent Asylum treated 57 cases last year; the President states with pride that the spirit of benevolence is developing amongst the wealthy dwellers at Port Fairy. The thirty-second annual report of St. Vincent's Hospital at Sydney, tells of 1,125 cases treated and a mortality of 7·60 per cent. Dr. J. J. Egan is resident physician and Mr. Charles D. McCarthy resident surgeon. The hospital is under the charge of the Sisters of Charity, and they have lately extended its departments by a hospice for chronic cases and a branch for phthisical patients.

Mr. Arthur Hulme also gave evidence as to the Queen's Hospital. He described the system of the admission of out-patients by registration at the hospital, and said that if a man had a wife and four children and received 35s. a week he was considered a suitable object for treatment. The registration fee of one shilling entitled a patient to attendance for six weeks. Enquiries were made as to the age of the applicant, the number of the family, the employment, and the wages received. Sometimes, when he was dissatisfied with the answers, further enquiries were made. In one week, out of 239 cases registered, seven were rejected as being applicants who were able to pay for advice. Mr. Hulme denounced the ticket system.

Considerable sorrow was felt in Chester last week when the death of Dr. Edward Waters became known, for he was one of the most brilliant of northern physicians. He was President of the British Medical Association in 1866, on the occasion of its annual meeting in Chester, and some four years ago was the recipient of that Association's distinguished merit medal—the third ever awarded—for his conspicuous labours in connection with securing the direct representation of the medical faculty on the Medical Council. The Lancashire and Cheshire branch of the British Medical Association about the same time testified by a presentation its high appreciation of Dr. Waters's services in the cause of medical reform, and this was followed by the public presentation of his portrait in Chester Town Hall, subscribed for by his professional friends and private admirers in and around the ancient city. He was a magistrate for the city of Chester, and honorary consulting physician to the Infirmary. Until recently he had enjoyed good health, and his death, which was painfully sudden, is attributed to failure of the heart's action.

The following vacancies are announced this week, the amount of the salary in each case being given after the name of the institution:—

**Resident Medical Officers.**

The Hospital for Sick Children, Great Ormond Street, House Surgeon—£50, board, &c.
Liverpool Dispensaries, Two Assistant Surgeons—£80, board, &c.
Borough Hospital, Sheffield, Medical Officer—£200, board, &c.
Manchester Royal Infirmary, Medical Officer—£150, board, &c.
Monsall Fever Hospital, Assistant Medical Officer—£100, board, &c.
Devon and Exeter Hospital, House Surgeon—£120, board, &c.
Bolton Infirmary and Dispensary, Senior House Surgeon—£120, board, &c.
Dorset County Asylum, Assistant Medical Officer—£135, board, &c.
Denbighshire Infirmary, House Surgeon—£85, board, &c.

**Non-Resident Medical Officers.**

London Hospital, Surgical Registrar—£100.
St. John's Hospital, Leicester Square, Assistant Physician.
Edinburgh Deaf and Dumb Institute, Oculist and Aurist.
Lincoln Oddfellows' Institute, Assistant Medical Officer—£104.
Bradford Friendly Societies, Medical Officer.
Dublin University, Examiners in Chemistry and Materia Medica.
Manchester Friendly Societies, Surgeon—£150.

A small home has been opened in Beckenham, the object being to assist clergy and others in a similar position of life to procure change of air for their delicate children, which, from pecuniary reasons, is in many cases an impossibility. Each child is kept for three weeks, the only expenses incurred by the parents being return fare and laundress. Girls are eligible between the ages of five and fourteen, boys between five and eight.

The Edinburgh Medical Missionary Society issues a very neat quarterly paper, which is specially interesting this time, because it contains a portrait and life of Mr. Benjamin Bell, F.R.C.S.E., grandson of the celebrated author of the *System of Surgery*. Accounts are given of work in Paris, Nazareth, Scutari, India, China, and British Columbia. The society trains its own students, and sends them forth to heal body and soul. The annual sale of work in connection with the society takes place on the 26th and 27th inst. at Edinburgh.

Two of the King's College medical staff have gone to Berlin to report on Dr. Koch's discoveries, and the senior surgeon of the Brompton Cancer Hospital has written for a supply of this new remedy for tuberculosis. The *Globe* is a very wicked little paper; it says that Koch will materially lower the death-rate of foreign countries by attracting all doctors to Berlin; but this style of joke is rather stale, and does not cause a laugh. The excitement in foreign medical circles is tremendous, and Koch's consumption cure is the only subject of conversation. Patients and medical men fill the Berlin hotels, and the frivolous globe-trotter can find no rest for the sole of his foot.

Dr. Jackson, for 25 years a general practitioner in Birmingham, has been giving evidence before the Hospital Reform Inquiry. He said he thought the hospitals were subject to abuse. The Chairman: If people can go to the hospital and receive first-rate advice and medicine, you don't blame them, do you; you blame the system? Dr. Jackson said he did blame them, because they ought to be ashamed of themselves for going if they could afford to pay. He considered the Hospital Saturday movement had great influence on the people, because they thought they had a right to go to the hospital, inasmuch as they paid a penny a week to the Hospital Saturday Fund. If, instead of contributing to the fund, working men were to subscribe and form a dispensary of their own it would relieve the hospitals very much, and provide employment for many medical men. In answer to Dr. Rickards, he said the misuse of the medical charities was chiefly and almost exclusively in the out-patient department.

